# Coexistence of gastrointestinal stromal tumor (GIST) and colorectal adenocarcinoma: A case report

**DOI:** 10.1186/1477-7819-5-96

**Published:** 2007-08-21

**Authors:** Papalambros Efstathios, Petrou Athanasios, Ioannis Papaconstantinou, Papalambros Alexandros, Sigala Frangisca, Georgopoulos Sotirios, Felekouras Evangelos, Giannopoulos Athanasios

**Affiliations:** 1 First Department of Surgery, Athens Medical School, National and Kapodistrian University of Athens, LAIKO General Hospital, Greece

## Abstract

**Background:**

Gastrointestinal stromal tumors (GIST) represent the most common mesenchymal tumors of the digestive tract. Over the last ten years the management of GISTs has dramatically altered but their coexistence with other gasrointesinal tumors of different histogenesis presents a special interest. The coexistence of GISTs with other primaries is usually discovered incidentally during GI surgery for carcinomas.

**Case presentation:**

We present here, a case of a 66-year-old patient with intestinal GIST and a synchronous colorectal adenocarcinoma discovered incidentally during surgical treatment of the recurrent GIST. Immunohistochemical examination revealed the concurrence of histologically proved GIST (strongly positive staining for c-kit, vimentin, SMA, and focal positive in S-100, while CD-34 was negative) and Dukes Stage C, (T3, N3, M0 according the TNM staging classification of colorectal cancer).

**Conclusion:**

The coexistence of GIST with either synchronous or metachronous colorectal cancer represents a phenomenon with increasing number of relative reports in the literature the last 5 years. In any case of GIST the surgeon should be alert to recognize a possible coexistent tumor with different histological origin and to perform a thorough preoperative and intraoperative control. The correct diagnosis before and at the time of the surgical procedure is the cornerstone that secures the patients' best prognosis.

## Background

Gastrointestinal stromal tumors (GISTs) are specific mesenchymal tumors that may occur anywhere along the gastrointestinal tract [1,2]. It is currently defined as gastrointestinal tract mesenchymal tumor containing spindle cells (less commonly epithelioid cells or both) and showing CD-117 (c-kit protein) and vimentin positivity [1,3,4]

GISTs are rare neoplasms and represent the 0.1–3% of all gastrointestinal cancers and the most common mesenchymal tumor of the digestive tract (20% of small bowel malignancies & approximately 5% of all tissue sarcomas) [5,6].

Over the last years the management of GISTs has evolved rapidly. A lot of changes have been reported in histological diagnostic criteria, in understanding of GISTs' molecular biology and pathogenesis, in imaging strategy, in surgical and adjuvant treatment etc [7]. Although the outcomes of several published series helped in understanding their pathogenesis, little is known about their coincidence with other tumors of different histogenesis. There are some data regarding the co-occurrence, the association and the potential common origin (genetic pathways of tumorigenesis), between GIST and other tumors [7,8]. The limited number of these cases can not confirm the existence of a common factor in tumorigenesis of these histopathologically completely different tumors and further studies are needed to clarify the possible association [9].

The coexistence of GISTs with other primaries is usually discovered incidentally during GI surgery for carcinomas [10-12]. Here in, we describe the reverse of the common situation, as a cecal adenocarcinoma was incidentally discovered during surgery for recurrent intestinal GIST.

## Case presentation

A 66-year-old man was admitted to our clinic two years ago complaining abdominal discomfort associated with distension, pain, and symptoms related to small intestine bowel obstruction (vomiting and obstipation). On physical examination a palpable mass in the lower right abdomen was found. CT scan of the abdomen and pelvis demonstrated a large (9 × 6.5 × 7 cm) low-density, well circumscribed mass in the right lower abdomen, without evidence of tumor infiltration of adjacent structures and without free fluid. No metastatic nodules were found in the liver and the lung.

Laparotomy revealed a pedunculated, ulcerative and friable large mass (a small bowel mass greater than 10 cm) with the features of gastroimtestinal stromal tumor: the surrounding organs (sigmoid colon, rectum, ureters and bladder) were pushed but not involved by the tumor. Furthermore, remarkable intraabdominal metastatic spread consisted of multiple small nodular lesions – less than 2 cm in size – all over the peritoneal surface and between bowel loops, was encountered. No evidence of liver metastasis or lymphadenopathy was found. The patient underwent an en-block resection of the tumor along with all visible disease in order to avoid capsule rupture and intraabdominal spillage, as is recommended [7]. A small bowel resection with an end to end primary anastomosis was performed. Numerous peritoneal nodal metastases were excised and sent for histological analysis.

Histopathological examination of the resected specimens revealed a stromal cell neoplasm with necrotic and hemorrhagic areas and a high index of mitotic count: 8–10 mitoses per high-power fields (HPF). Immunohistochemical analysis revealed C-kit (strongly positive), SMA(strongly positive), focal S-100(+) and CD34(-). The result of histological examination confirmed also the existence of intraabdominal metastatic spread.

Following the histopathological confirmation, immediate treatment with imatinib was initiated. The imatinib was introduced in dose of 400 mg/day without interruption (in order to minimize the risk of relapse associated with the drug interruption) [10]. Postoperatively, patient was followed up with CT scan. According to the clinical, surgical and histopathological features, our patient was included in the high risk group for recurrence. The patient underwent a CT scan of the abdomen and the pelvis in regular 4–6 months intervals [13,14].

**Figure 1:**
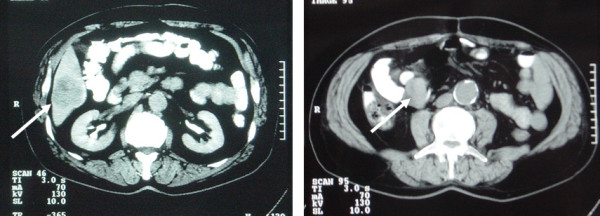
A: CT scan of the abdomen revealing, A: a 5 × 5.2 cm liver mass (white arrow) located between segments V and VI and B: a 4 × 3.8 cm soft tissue mass (white arrow) in the right abdomen between bowel loops.

The CT scan performed 2 years after surgical excision and treatment commencement, revealed metastatic liver disease. Dose escalation (400 mg of imatinib mesylate twice a day) was decided and initiated. Six months later, liver lesion was presented with a characteristic size increase (figure 1a). Furthermore, a small soft tissue mass (3,3 × 2,8 × 3 cm) in the right lower abdomen was revealed in the abdominal CT scan. The mass was localized at the anatomic area of the previous excision and was considered either as another metastatic lesion or as a possible local recurrence (figure 1b).

Laparotomy confirmed the presence of liver metastasis, localized between the Segment V & Segment VI as well as intra-abdominal metastatic spread (multiple small nodular lesions – less than 1 cm in size – all over the peritoneal surface and between bowel loops). An intestinal mass was also found 60 cm proximal to the ileocecal valve. Surprisingly, during the thorough exploration of the peritoneal cavity, another mass with completely different macroscopic features was discovered. It was a palpable cecal mass, without extracanalicular growth or infiltration into other organs, associated with local lymph-node enlargement.

Intra-abdominal ultrasound (IOUS) was performed in order to assist in liver resection planning (mainly to enable detection of additional tumors, missed in preoperative CT scan imaging and to evaluate the relationship between metastatic lesions and major vascular structures).

RFA (Radio Frequency Ablation)-assisted liver resection [15] of segments V, VI was performed and a segmental small-bowel resection along with excision of all gross visible peritoneal nodular lesions followed. Finally, a right hemicolectomy was decided in order to treat the "unexpected" cecal lesion.

At this point is important to underline that surgery remains the mainstay therapy for both (GIST and colorectal cancer) although the operative strategies and extent of resection are fairly different. [12] Given the rarity of lymph-node involvement, routine lymphadenectomy is not currently recommended in GIST cases. [13,16] At the present case because of local lymph-node enlargement a curative resection was performed (included the resection of the lymphatic station), aiming to obtain microscopic disease-free radial and distal margins.

Gross examination of the 29 cm specimen disclosed a 7 × 4 cm tumor originating from the cecum and two polyps located about 7 cm from the cecum. The liver specimen (8.5 × 6.5 × 3.7 cm, segments V, VI) weighted 90 gr and contained the 5 cm metastatic lesion.

**Figure 2:**
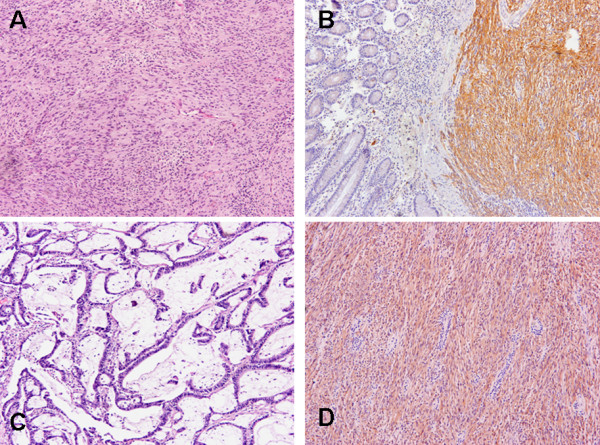
A: Small intestine GIST in H-E stain (×20); B: C-kit immunoexpression (×40); C: Adenocarcinoma of cecum in H-E stain (×20); D: C-kit immunoreactivity in metastatic GIST of liver (×20).

Histopathological examination of the resected specimen (figure 2) revealed a recurrent intestinal GIST (figure 2A,B) with malignant biological behavior and a moderately differentiated stage Dukes C (T3, N3, M0 according the TNM staging classification of colorectal cancer) cecal adenocarcinoma (figure 2C).

The specimen had the following characteristics: a) metastatic liver tumor with mesenhymal origin characterized by necrotic and bleeding areas (figure 2D) and a mitotic count of <5/50 HPF. Immunohistochemical staining revealed a strongly (+) reaction for c-kit, slight (+) for S-100, and no specific staining for CD34 and desmin. The surgical margins of the resected hepatic specimen were free from invasion, b) moderately differentiated adenocarcinoma with a slight extracellular, mucus production. The tumor invaded the muscularis propria and the pericolonic adipose tissue. The neoplastic tissue extended and infiltrated the small bowel wall but the proximal and distant surgical margins were free.

Four of the examined lymph nodes presented metastatic invasion from GIST and 12 from colorectal adenocarcinoma. The above mentioned morphologic and immunochistochemical findings are diagnostic for coexistence of GIST and colorectal adenocarcinoma

## Discussion

Gastrointestinal stromal tumors are uncommon and their pathogenesis, diagnosis, nomenclature, and prognosis have long been a subject of considerable controversy. The stromal tumors designated as GISTs are identified by a series of histological, molecular, genetic and in particular, immunophenotypic features that set them apart from common leiomyomas, leiomyosarcomas of the GI-tract. Recent studies suggest that GISTs represent a subgroup of gastrointestinal mesenchymal tumors arising from a common precursor cell, the interstitial cell of Cajal, or a primitive stem cell from which both Cajal cells and smooth muscle cells arise [3]. Gastrointestinal stromal tumors are currently defined as c-kit (CD117)-positive spindle cell (or, less commonly, epithelioid cell or both) gastrointestinal tract mesenchymal tumors [1,3].

The most important markers for defining GISTs are CD117 (c-kit protein) and CD 34 (hematopoietic cell progenitor antigen). The majority of GISTs are usually positive for CD117 (near 95% of cases), CD34 (positive in 70–80% of cases), smooth muscle actin (positive in 40% of cases), PS 100 (positive near 5% of cases), and desmin (positive in approximately 2% of cases) [1-3].

Although the demonstration of c-kit (CD117) immunoreactivity represents the gold standard for diagnosis of GIST (it's also considered as an eligibility criterion for Imatinib Masylate therapy) [2,3] approximately 5% of histologocally suspected GIST are CD117 negative [1,17,18].

Gastrointestinal stromal tumors may occur anywhere along the digestive tract. They may be found anywhere between the esophagus and the anus. Additional locations have been found to include omentum, mesentery and retroperitoneum [18,19,21]. Most GISTs arise from the stomach (50–62%), from the small intestine (20–30%), the colon (11%), the rectum (7%) while the esophagus is rarely involved (0,6–1%) [19,20,22].

It is well known that GISTs have the potential to metastasize (at first diagnosis many patients present with metastatic spread (11–47%) [22] either via blood stream or via peritoneal seeding and they give rise to liver or omental metastases. Lung, bones and lymph node metastases are rare [1,2]. GISTs' malignant potential is considered to depend on tumor size, frequency of mitoses, presence of necrosis, and the possible invasion to adjacent organs [21-24] but there is also a relevance to the anatomic site of origin [3].

In the described case, a 66-year-old male was operated primarily for a small bowel tumor in surgical emergency setting. The tumor was treated as malignant because, according to the guidelines, in a surgical emergency or in the absence of a preoperative diagnosis, even in the presence of a single mass without evidence of contiguous organ involvement or metastatic disease, the surgeon is responsible to recognize and to treat these lesions as malignant [25]. Histopathologic findings confirmed the presence of intestinal GIST with a highly malignant behavior. Reported series in pre-imatinib era indicated that the complete surgical excision, although technically feasible, is not curative and the use of imatinib in unresectable and/or metastatic disease is recommended [13]. Indeed, the patient was further treated with imatinib mesylate and underwent the indicated by ESMO follow up.

Two years after treatment initiation, during follow-up CT scan, a liver mass was found. The liver mass failed to respond to the appropriate imatinib dose escalation (400 mg twice day). Additionally, at the subsequent follow up, an intestinal mass at the site of previous surgical excision was discovered, which was initially confused with other similar metastatic lesions demonstrated between small bowel loops.

Surgery followed and the subsequent histopathological examination confirmed the presence of the GIST liver metastasis and a moderately differentiated cecal adenocarcinoma. a fact that renders the potential promising therapeutic impact of imatinib mesylate in the prevention of colorectal cancer controversial (this was shown by the inhibition of the in vitro growth of the colorectal carcinoma cell lines) [26].

There is a continuously increasing knowledge about synchronous presentation of GIST and other gastrointestinal tumors. The major types of GIST-associated malignancies reported in literature are: gastrointestinal carcinomas (gastric and colon cancer), lymphoma/leukemia, gynecological carcinomas, and carcinomas of prostate, breast, pancreas, lung, liver, kidney as well as carcinoid of pancreas and stomach [10-12,27-30]. Although uncommon, the fact that patients who have a history of soft tissue sarcomas (STS) are in an increased risk for the development of GIST, presents a special interest [30,31].

Even if the synchronous occurrence of GIST and colorectal adenocarcinomas is not extremely rare little is yet known their potential common origin and carcinogenetic pathways. The limited number of reported cases cannot rule out a possible incidental occurrence though [32,33]. The majority of the coexistent GISTs are discovered incidentally during work-up or during therapeutic procedures for GI malignancies. What is unique in the present case is that synchronous cecal adenocarcinoma was incidentally discovered during laparotomy for recurrent GIST disease.

What should be delineated at this point is the undeniable possible coexistence of a synchronous tumor with a primary or recurrent GIST. This fact underlines the significance of a detailed preoperative imaging study which should include contrast enhanced CT scans of the abdomen and pelvis. Other modalities used in the evaluation of GISTs include MRI and Fluorodeoxyglcose positron emission tomography (PET) but CT is currently the imaging modality of choice. Above all, we should emphasize the importance of a thorough intrabdominal exploration by an experienced surgeon in order not to overlook a possible mass that could hide a synchronous cancer.

## Conclusion

The coexistence of GIST with either synchronous or metachronous colorectal cancer represents a phenomenon with increasing number of relative reports in the literature the last 5 years.

In any case of GIST (with or without preoperative histopathological confirmation) the surgeon should be alert to recognize a possible coexistent tumor with different histological origin and to perform a thorough preoperative and intraoperative control. The correct diagnosis before and at the time of the surgical procedure is the cornerstone that secures the patients' best prognosis.

## Competing interests

The author(s) declare that they have no competing interests.

## Authors' contributions

PE: author and supervisor of the manuscript.

PA: editing.

PI: editing.

PA: collection of the literature.

SF: collection of the literature.

GS: revision of the manuscript.

FE: revision of the manuscript.

GA: supervisor of the manuscript and final revision.
